# Preoperative gravity stress radiograph parameters predicting the need for deltoid ligament repair after lateral malleolar fixation in Weber B ankle fractures

**DOI:** 10.1186/s13018-026-06915-z

**Published:** 2026-05-02

**Authors:** Jaehyung Lee, Jihun Park, Jae Yong Park

**Affiliations:** 1 Department of Orthopaedic Surgery, Seoul Now Hospital, Anyang-si, Gyeonggi-do Republic of Korea; 2 Department of Orthopaedic Surgery, Busan Paik Hospital, Inje University College of Medicine, Busan, Republic of Korea; 3 Department of Orthopaedic Surgery, Hallym University Sacred Heart Hospital, Hallym University College of Medicine, Anyang-si, Gyeonggi-do Republic of Korea

**Keywords:** Ankle fracture, Deltoid ligament, Gravity stress radiograph, Medial clear space, Medial clear space angle

## Abstract

**Background:**

Radiographic criteria for deltoid ligament repair in Weber B ankle fractures without a medial malleolar fracture remain unclear. Medial clear space (MCS) on gravity stress radiographs is commonly used to assess medial instability, but the optimal cutoff value and diagnostic accuracy are still debated.

**Methods:**

This retrospective comparative study included 55 patients with Weber B ankle fractures without medial malleolar fractures who underwent operative treatment between 2016 and 2021. All patients underwent preoperative gravity stress radiography and intraoperative fluoroscopic stress testing after completion of fracture stabilization. Patients were classified as unstable (deltoid ligament repair due to persistent medial instability) or stable (no repair). Preoperative radiographic parameters—including MCS, medial clear space angle (MCA), superior clear space (SCS), medial-to-superior clear space ratio, fibular lateral shift distance (FLSD), and MCS–FLSD difference—were measured. Diagnostic performance and optimal cutoff values were evaluated using receiver operating characteristic curves and Youden’s index.

**Results:**

Sixteen patients were classified as unstable and 39 as stable. On preoperative gravity stress radiographs, the unstable group showed significantly greater MCS (8.3 ± 2.6 vs. 5.9 ± 1.9 mm, *p* = 0.002) and MCA (16.6 ± 3.8° vs. 6.4 ± 3.1°, *p* < 0.001) than the stable group. MCA demonstrated high discriminative ability, with an area under the curve of 0.98, whereas MCS showed an area under the curve of 0.81. The optimal cutoff values were 6.0 mm for MCS and 11.5° for MCA. At 1 year postoperatively, clinical outcomes were similar between groups.

**Conclusions:**

MCA demonstrated high discriminative ability on preoperative gravity stress radiographs and may serve as a useful adjunct to MCS for anticipating the need for deltoid ligament repair after completion of fracture stabilization in Weber B ankle fractures.

## Background

Ankle fractures are among the most common injuries encountered in orthopaedic practice, and trans-syndesmotic ankle fractures (Weber type B) are the most frequent pattern [1, 2]. Because most Weber B fractures result from rotational forces, injury to the medial structures—such as a medial malleolar fracture or a deltoid ligament rupture—is common [3, 4]. In the absence of a medial malleolar fracture, accurate assessment of clinically relevant medial instability is important, as unrecognized medial instability can lead to chronic ankle instability and post-traumatic arthritis [5, 6].

The deltoid ligament complex consists of superficial and deep components. The superficial deltoid primarily resists hindfoot eversion, whereas the deep deltoid is the primary restraint to external rotation of the talus relative to the tibia [7, 8]. Experimental and clinical studies have shown that incompetence of the deep deltoid ligament can be associated with lateral talar shift, widening of the medial clear space (MCS), and loss of mortise congruency, even when the fibula is anatomically reduced [9–11]. Therefore, reliable preoperative assessment of clinically relevant deltoid ligament injury remains an important issue when treating Weber B ankle fractures with an intact medial malleolus [3, 6, 12].

When deltoid ligament injury is suspected on physical examination but is not evident on standard radiographs, additional imaging modalities—such as magnetic resonance imaging, ultrasound, and stress radiography—may be used to assess medial stability [3, 12]. Among these, gravity stress radiographs have gained popularity because they are simple, inexpensive, and relatively well tolerated, and can be obtained without manual manipulation [12–14]. On gravity stress views, several radiographic parameters—including MCS, superior clear space, and side-to-side differences compared with the contralateral ankle—have been proposed to define medial instability [10, 12, 14, 15].

Traditionally, increased MCS on stress radiographs has been used as an indirect marker of deep deltoid ligament rupture, with cutoffs between 4 and 5 mm often cited to indicate instability in supination–external rotation or Weber B fractures [10, 12, 16]. However, the optimal cutoff value remains controversial, and prior studies have reported substantial variability in “normal” MCS as well as inconsistencies between physical examination, stress radiograph findings, and the actual status of the deltoid ligament [10, 12, 14–17]. Furthermore, MCS is influenced by ankle position, the type and magnitude of applied stress, and lateral talar shift secondary to fibular displacement, which may limit its specificity for deep deltoid injury [4, 9, 11, 14]. Clinically, some patients with positive preoperative stress radiographs show resolution of medial joint space widening after lateral malleolar fixation alone, suggesting that not all cases of MCS widening require deltoid ligament repair [3, 6, 12].

At the same time, the role of deltoid ligament repair (DLR) in acute ankle fractures remains debated. Recent systematic reviews have suggested that DLR may improve radiographic parameters such as postoperative MCS and reduce malreduction rates, although a consistent functional advantage has not been established [6, 18]. In many centers, the deltoid ligament is explored or repaired selectively, typically when there is a block to reduction or when residual medial instability persists after completion of fracture stabilization [3, 6, 19]. In this context, preoperative radiographic parameters that help anticipate persistent medial instability and the intraoperative need for deltoid ligament repair may be useful for surgical planning and patient counselling.

Because the deep deltoid ligament is an important restraint to talar external rotation, an angular radiographic parameter may better reflect the rotational component of medial instability than linear joint-space measurements alone [8, 9, 11, 16]. Based on this concept, we defined the medial clear space angle (MCA) as the angle between the medial articular surface of the talus and the lateral articular surface of the medial malleolus on the gravity stress mortise view.

The purpose of this retrospective comparative study was to compare preoperative gravity stress radiographic parameters between patients who demonstrated persistent medial instability after completion of fracture stabilization and those who did not, and to identify radiographic parameters associated with the intraoperative need for deltoid ligament repair in selected Weber B ankle fractures without a medial malleolar fracture.

## Methods

### Study design and patient selection

This retrospective comparative study included patients treated at our institution between 2016 and 2021. Patients were eligible if they had a Weber B–type ankle fracture without a concomitant medial malleolar fracture, underwent operative treatment with lateral malleolar fixation, and had a minimum clinical follow-up of 12 months. At presentation, all patients underwent standard ankle radiographs followed by a physical examination. Deltoid ligament injury was suspected in the presence of medial tenderness, point tenderness, or swelling on the medial side of the ankle [6].

**Fig. 1:**
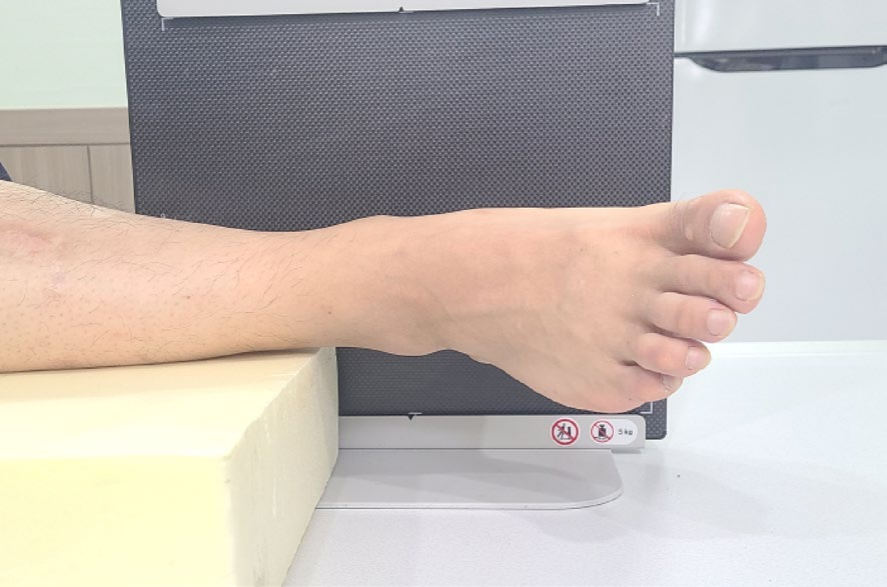
Technique for obtaining the gravity stress radiograph

When deltoid ligament injury was suspected clinically, a gravity stress radiograph of the injured ankle was obtained and compared with the initial radiographs. Gravity stress radiographs were obtained in the radiology room with the patient lying on the side, the injured limb dependent. The ankle joint was positioned beyond the edge of the table, and the ankle was internally rotated approximately 10° to obtain an appropriate mortise projection with the X-ray beam centered on the ankle and directed toward the cassette (Fig. 1). Patients with an MCS greater than 4 mm on the gravity stress radiograph were considered to have potentially unstable fractures and were scheduled for operative fixation of the lateral malleolus with possible deltoid ligament repair [12, 14].

Patients were excluded if they had concomitant ipsilateral tibial fractures, bilateral ankle fractures, pediatric fractures (skeletally immature), or inadequate radiographs that precluded accurate measurement of the radiographic parameters. After application of the inclusion and exclusion criteria, 55 patients were available for analysis.

### Surgical procedure and intraoperative assessment

All operations were performed under general or spinal anesthesia by a single senior orthopaedic surgeon (J.Y. Park). In the operating room, hematoma evacuation and open reduction and internal fixation of the lateral malleolus were first performed using a plate and screws. In cases with an associated posterior malleolar fracture, fixation was performed selectively when fragment size, morphology, displacement, and articular incongruity suggested that stabilization of the posterior fragment would contribute to restoration of joint congruity. When indicated, the posterior malleolar fragment was fixed with a posterior-to-anterior screw.

After completion of fibular fixation and, when indicated, posterior malleolar fixation, syndesmotic stability was assessed intraoperatively using the Cotton test under fluoroscopy. If residual syndesmotic instability was identified, trans-syndesmotic fixation was performed using one or two cortical screws.

Medial stability was assessed only after all required bony and syndesmotic fixation had been completed. With the patient in the supine position, manual external rotation and valgus (abduction) stress tests were applied under real-time fluoroscopy, and mortise views of the ankle were obtained. Persistent medial instability was defined primarily as medial clear space widening greater than the superior clear space on stress fluoroscopy [12, 20, 21]. In equivocal cases, the contralateral ankle was used as a reference when available; when contralateral imaging was not feasible, the final judgment was made by the senior surgeon based on the overall fluoroscopic appearance and clinical findings.

**Fig. 2:**
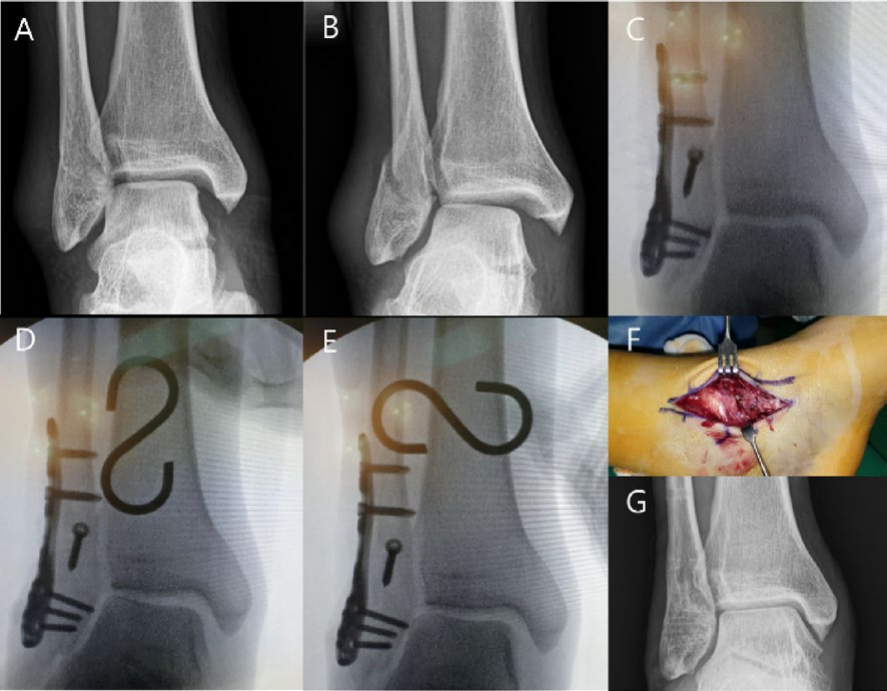
Representative radiographs and an intraoperative photograph of a 49-year-old man with a lateral malleolar fracture. **A** Preoperative anteroposterior ankle radiograph. **B** Preoperative gravity stress radiograph. **C** Intraoperative fluoroscopic image after lateral malleolar fixation. **D** Intraoperative valgus stress view. **E** Intraoperative external rotation stress view. These stress views demonstrated persistent medial instability after fixation; therefore, deltoid ligament repair was performed. **F** Intraoperative gross photograph showing rupture of the deep deltoid ligament. **G** Standing anteroposterior ankle radiograph obtained 13 months postoperatively

**Fig. 3:**
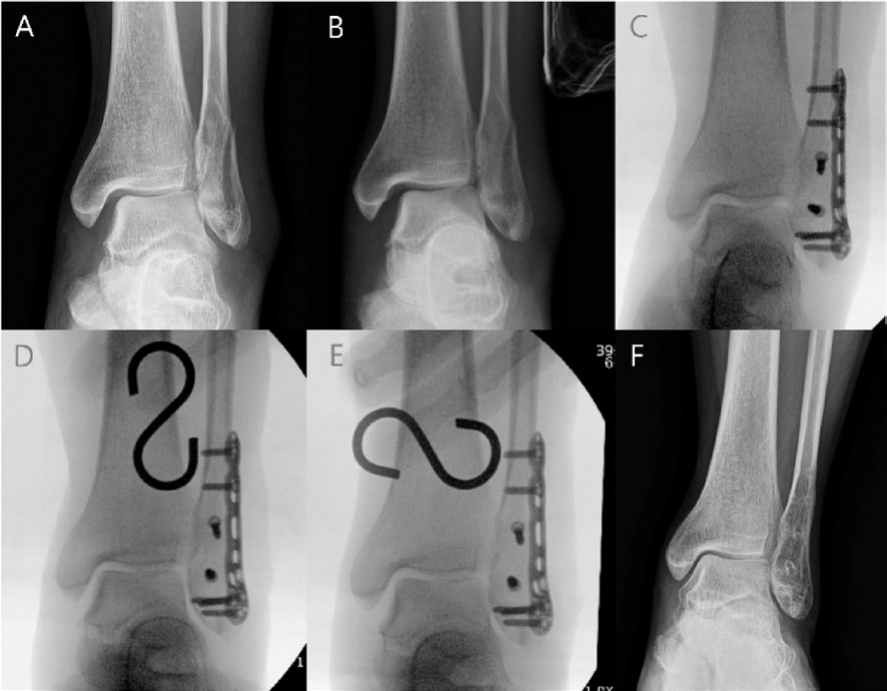
Representative radiographs of a 54-year-old woman with a lateral malleolar fracture. **A** Preoperative anteroposterior ankle radiograph. **B** Preoperative gravity stress radiograph. **C** Intraoperative fluoroscopic image after lateral malleolar fixation. **D** Intraoperative valgus stress view. **E** Intraoperative external rotation stress view. These stress views showed no medial instability after fixation; therefore, deltoid ligament repair was not performed. **F** Standing anteroposterior ankle radiograph obtained 14 months postoperatively

Patients with persistent medial instability after completion of bony and syndesmotic stabilization underwent deltoid ligament repair and were classified as the unstable group (Fig. 2), whereas those in whom medial stability was restored without deltoid repair were classified as the stable group (Fig. 3).

For deltoid ligament repair, a medial incision was made to expose the deltoid ligament while carefully protecting the neurovascular structures. The superficial and deep components of the deltoid ligament were inspected. When a deep deltoid ligament rupture was identified, it was repaired using a suture anchor or nonabsorbable sutures (Ethibond; Ethicon Inc., Somerville, NJ, USA). The superficial deltoid ligament was repaired with absorbable sutures when the tissue quality allowed for repair.

### Radiological and clinical evaluation

Radiological evaluation was performed using standard plain radiographs obtained at four time points: preoperative gravity stress radiographs, preoperative non–weight-bearing radiographs, immediate postoperative radiographs, and radiographs at the final follow-up. All radiographs were obtained in a standardized manner according to institutional protocols.

Two independent orthopaedic residents, blinded to the intraoperative group allocation, measured the radiographic parameters on digital images using a picture archiving and communication system. Each observer performed the measurements twice at least 2 weeks apart, and the mean of the two measurements was used for analysis. Inter- and intra-observer reliability were assessed using intraclass correlation coefficients.

**Fig. 4:**
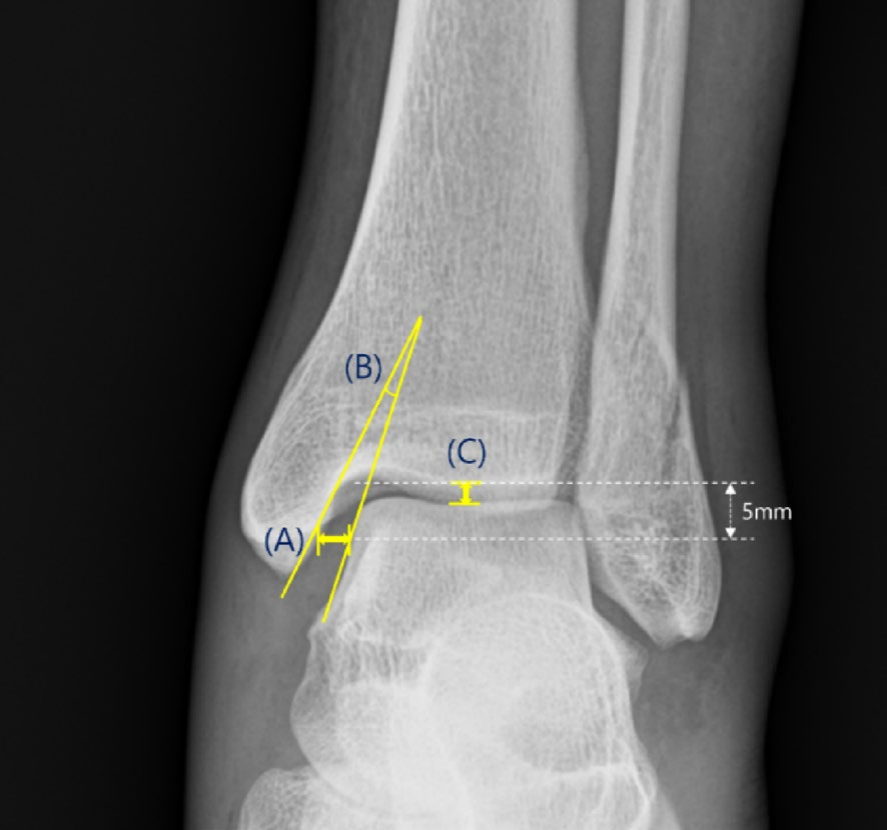
Preoperative radiographic measurements on gravity stress views. **A** Medial clear space (MCS) is measured as the distance between the medial border of the talus and the lateral border of the medial malleolus, 5 mm below the tibial plafond. **B** Medial clear space angle (MCA) is defined as the angle formed by the medial articular surface of the talus and the lateral articular surface of the medial malleolus. **C** Superior clear space (SCS) is measured as the distance between the superior articular surface of the talus and the tibial plafond

Measured radiographic parameters on the preoperative gravity stress mortise view included MCS, MCA, superior clear space (SCS), medial–superior clear space ratio (M/S ratio), and fibular lateral shift distance (FLSD). MCS was defined as the distance between the medial border of the talus and the lateral border of the medial malleolus, measured 5 mm below the tibial plafond and parallel to the joint surface. MCA was defined as the angle between the medial articular surface of the talus and the lateral articular surface of the medial malleolus. SCS was measured as the distance between the superior articular surface of the talus and the tibial plafond at the center of the talar dome (Fig. 4). The M/S ratio was calculated as the ratio of MCS to SCS. FLSD was defined as the horizontal distance between the lateral edge of the distal fibular fragment and the medial edge of the proximal fibular fragment at the midpoint of the fracture line. In addition, the difference between the MCS on the gravity stress radiograph and FLSD (MCS–FLSD difference) was calculated as an exploratory adjusted measure [4, 15].

Clinical outcomes at follow-up were assessed using the visual analog scale (VAS) for pain, the Foot Function Index (FFI), the Foot and Ankle Outcome Score (FAOS), and the Olerud–Molander Ankle Score (OLERUD).

### Statistical analysis

Statistical analyses were performed using IBM SPSS Statistics (version 26.0; IBM Corp., Armonk, NY, USA). Statistical power was estimated using G*POWER software (version 3.1.9.2; Heinrich Heine Universität, Düsseldorf, Germany) based on the observed effect size of MCA between the stable and unstable groups. Continuous variables were tested for normality using the Shapiro–Wilk test. Depending on the distribution, differences in radiographic and clinical parameters between the stable and unstable groups were compared using independent t-tests or Mann–Whitney U tests. Categorical variables were compared using chi-square or Fisher’s exact tests as appropriate. A* p*-value < 0.05 was considered statistically significant.

The discriminative performance of MCS, MCA, and other radiographic parameters for anticipating persistent medial instability requiring deltoid ligament repair was evaluated using receiver operating characteristic (ROC) curves, and the area under the curve (AUC) was calculated with 95% confidence intervals. Optimal cutoff values were identified using Youden’s index. Sensitivity, specificity, positive predictive value, and negative predictive value were then calculated at the selected cutoffs.

Because of the limited number of unstable cases, adjusted analyses were restricted to exploratory models with MCA or MCS entered as the primary predictor and one clinically relevant covariate at a time, including syndesmotic fixation or posterior malleolar fracture. Exploratory sensitivity analyses were also performed after excluding patients who underwent syndesmotic fixation, those with posterior malleolar fractures, and those who underwent posterior malleolar fixation.

## Results

From an initial pool of 88 patients with Weber B–type ankle fractures without medial malleolar fractures, 5 patients with complex ipsilateral tibial and fibular fractures, 7 with bilateral ankle fractures, 4 with pediatric fractures, and 17 with inadequate radiographs were excluded. After application of the inclusion and exclusion criteria, 55 patients were included in the final analysis.

**Fig. 5:**
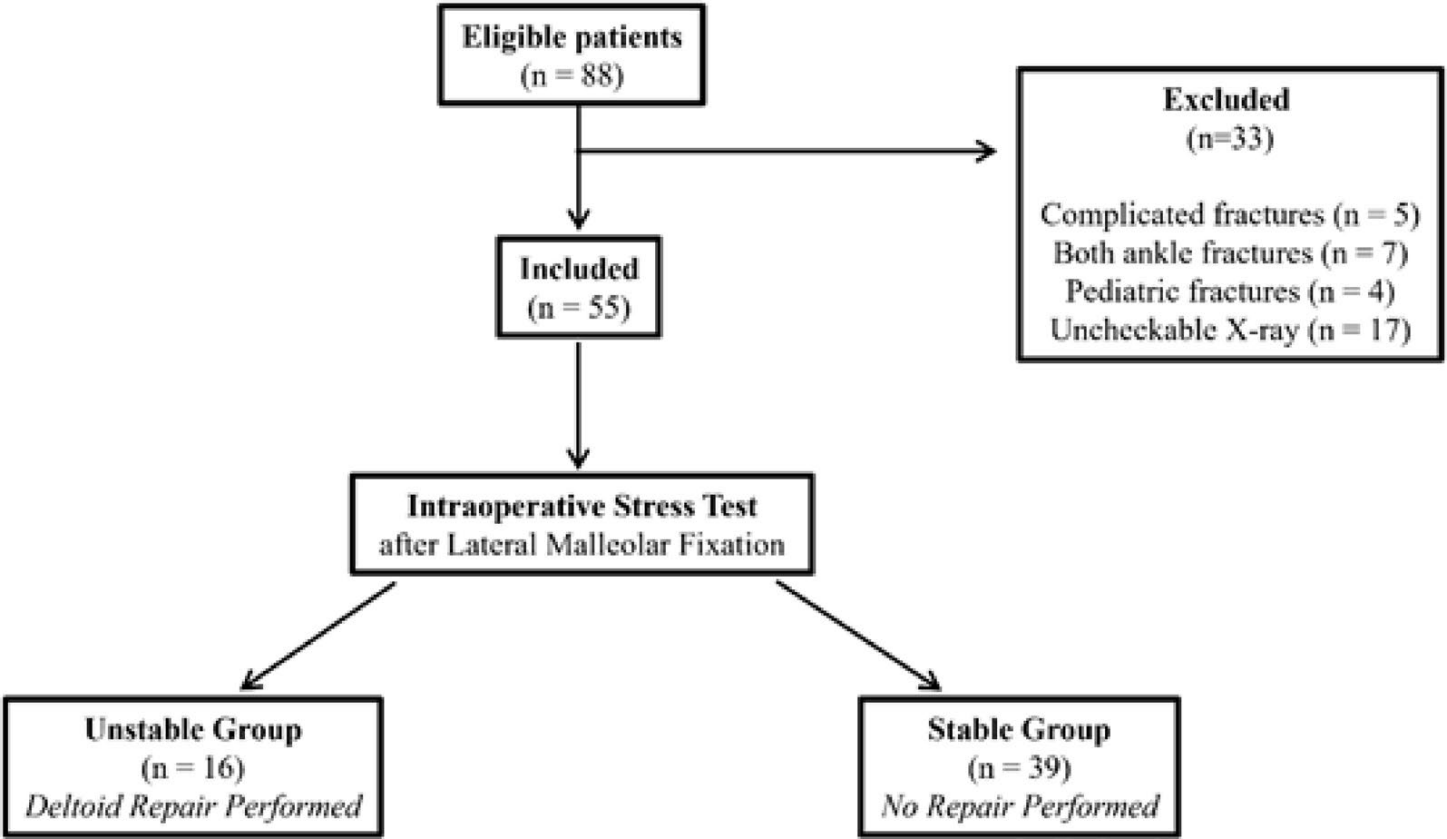
Detailed flowchart of patient selection and grouping

Based on intraoperative fluoroscopic assessment after completion of bony and syndesmotic stabilization, 16 patients were classified as the unstable group, in whom persistent medial instability required deltoid ligament repair, and 39 were classified as the stable group, in whom medial stability was restored without deltoid ligament repair (Fig. 5). The mean follow-up duration was 15.9 months (range, 12–51 months).

In the overall cohort of 55 patients, posterior malleolar fractures were identified in 29 cases, of which 6 underwent posterior malleolar fixation. There were no significant differences between the two groups in posterior malleolar fracture, posterior malleolar fixation, or syndesmosis fixation (Table 1).

**Table 1:** Comparison of associated fracture and fixation variables according to deltoid ligament repair

Variable	Stable (*n* = 39)	Unstable (*n* = 16)	*p*-value
Posterior malleolar fracture, n (%)	21 (53.8)	8 (50.0)	1.000
Posterior malleolar fixation, n (%)	4 (10.3)	2 (12.5)	1.000
Syndesmosis fixation, n (%)	2 (5.1)	2 (12.5)	0.571

Data are presented as number (%).* P*-values were calculated using Fisher’s exact test

Preoperative radiographic parameters on gravity stress views are summarized in Table 2. The unstable group showed significantly greater MCS (8.3 ± 2.6 vs. 5.9 ± 1.9 mm, *p* = 0.002), M/S ratio (1.9 ± 0.6 vs. 1.5 ± 0.5, *p* = 0.002), and MCA (16.6 ± 3.8° vs. 6.4 ± 3.1°, *p* < 0.001) compared with the stable group. In contrast, SCS (4.4 ± 0.6 vs. 4.3 ± 1.2 mm, *p* = 0.981) and MCS–FLSD difference (1.1 ± 1.0 vs. 1.2 ± 1.0 mm, *p* = 0.732) did not differ significantly between groups.

**Table 2:** Comparison of preoperative gravity stress radiographic parameters between stable and unstable groups

Parameter	Unstable group (*n* = 16)	Stable group (*n* = 39)	*p*-value
MCS (mm)	8.3 ± 2.6	5.9 ± 1.9	0.002
MCA (°)	16.6 ± 3.8	6.4 ± 3.1	< 0.001
M/S ratio	1.9 ± 0.6	1.5 ± 0.5	0.002
SCS (mm)	4.4 ± 0.6	4.3 ± 1.2	0.981
MCS–FLSD (mm)	1.1 ± 1.0	1.2 ± 1.0	0.732

MCS Medial clear space, MCA Medial clear space angle, M/S ratio Medial–superior clear space ratio, SCS Superior clear space, FLSD Fibular lateral shift distance

**Fig. 6:**
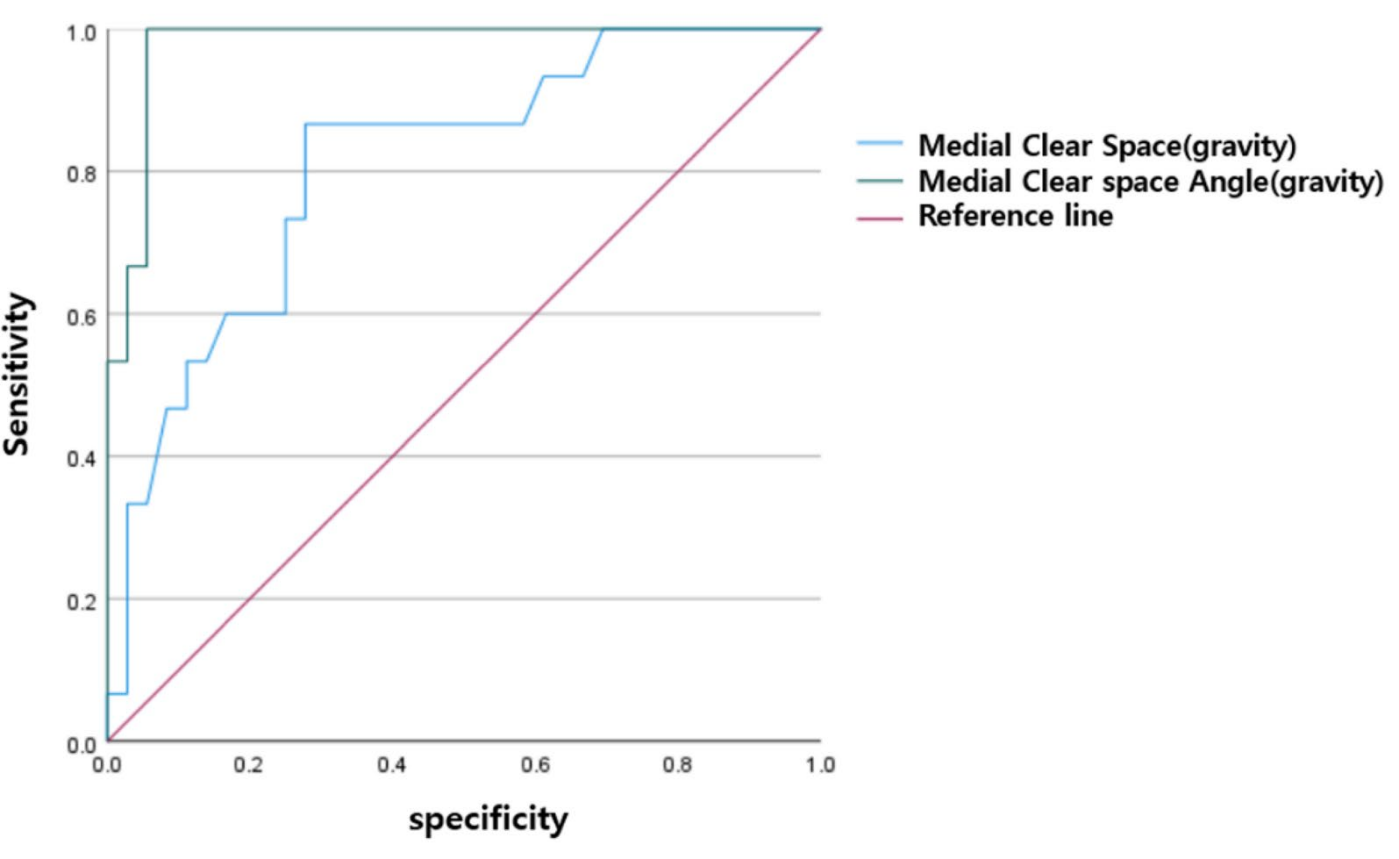
Receiver operating characteristic (ROC) curves for diagnostic parameters

Receiver operating characteristic curve analysis for anticipating persistent medial instability requiring deltoid ligament repair is shown in Fig. 6; Tables 3 and 4. MCA demonstrated a high discriminative ability, with an AUC of 0.98 (95% CI, 0.93–1.00), whereas MCS showed an AUC of 0.81 (95% CI, 0.68–0.94). The optimal cutoff value determined by Youden’s index was 11.5° for MCA, yielding a sensitivity of 94% and a specificity of 100%, and 6.0 mm for MCS, yielding a sensitivity of 88% and a specificity of 71%. Although MCA showed numerically greater discriminative performance than MCS, this difference should be interpreted cautiously given the limited sample size.

**Table 3:** Cutoff values and overall accuracy of radiographic parameters

Parameter	Optimal cutoff	AUC (95% CI)	Youden’s index
MCA (°)	> 11.5	0.98 (0.93–1.00)	0.94
MCS (mm)	> 6.0	0.81 (0.68–0.94)	0.59

AUC Area under the curve, CI Confidence interval, MCA Medial clear space angle, MCS Medial clear space

**Table 4:** Diagnostic performance (sensitivity, specificity, and predictive values)

Parameter	Sensitivity (%)	Specificity (%)	PPV (%)	NPV (%)
MCA	94	100	100	98
MCS	88	71	50	96

PPV Positive predictive value, NPV Negative predictive value, MCA Medial clear space angle, MCS Medial clear space

Exploratory adjusted analyses were performed to assess whether the association between MCA and persistent medial instability remained after limited adjustment for potential confounding factors. In a model including MCA and syndesmotic fixation, MCA remained significantly associated with persistent medial instability (odds ratio [OR], 1.53; 95% CI, 1.21–1.95; *p* < 0.001), whereas syndesmotic fixation was not significant (OR, 6.36; 95% CI, 0.19–211.11; *p* = 0.300). Similarly, in a model including MCA and posterior malleolar fracture, MCA remained significantly associated with persistent medial instability (OR, 1.65; 95% CI, 1.23–2.21; *p* < 0.001), whereas posterior malleolar fracture was not significant (OR, 0.20; 95% CI, 0.03–1.45; *p* = 0.111). MCS also remained significant in analogous exploratory models that included syndesmotic fixation or posterior malleolar fracture.

In subgroup-based sensitivity analyses (Table 5), the association between MCA and persistent medial instability remained present after exclusion of patients with syndesmotic fixation, posterior malleolar fractures, or posterior malleolar fixation.

**Table 5:** Exploratory sensitivity analyses after exclusion of potential confounding subgroups

Sensitivity analysis subset	Remaining cohort, *n* (stable/unstable)	Parameter	*p*-value	AUC
Excluding patients who underwent syndesmotic fixation	51 (37/14)	MCA	< 0.001	0.96
		MCS	0.002	0.78
Excluding patients with posterior malleolar fracture	26 (18/8)	MCA	< 0.001	0.99
		MCS	< 0.001	0.94
Excluding patients who underwent posterior malleolar fixation	49 (35/14)	MCA	< 0.001	0.97
		MCS	< 0.001	0.82

MCA Medial clear space angle, MCS Medial clear space, AUC Area under the receiver operating characteristic curve

*P*-values were calculated for comparisons between the stable and unstable groups within each restricted cohort. Sensitivity analyses were performed to assess whether the association between preoperative radiographic parameters and persistent medial instability remained after excluding potential confounding subgroups.

Clinical outcomes at 12 months postoperatively are summarized in Table 6. There were no significant differences between the unstable and stable groups in VAS pain scores, FFI, FAOS, or Olerud–Molander Ankle Scores (all *p* > 0.05). No patients in either group required revision surgery for loss of fixation, syndesmotic widening, or recurrent medial instability during the follow-up period.

**Table 6:** Comparison of clinical outcomes of the unstable group and the stable group

Variables	Unstable (*n* = 16)	Stable (*n* = 39)	*p*-value	
VAS	1.53	1.17	0.385	
FFI	4.74	3.96	0.502	
FAOS	83.70	87.57	0.352	
OLERUD	85.67	84.86	0.878	

VAS Visual analog scale, FFI Foot function index, FAOS Foot and ankle outcome score, OLERUD Olerud–molander ankle score

## Discussion

This study investigated whether preoperative gravity stress radiographic parameters could help anticipate persistent medial instability after fracture stabilization in Weber B ankle fractures without medial malleolar fractures. In this cohort, approximately one-third of patients with preoperative medial clear space (MCS) widening ultimately required deltoid ligament repair because of persistent medial instability after fracture stabilization, whereas the remaining patients achieved stability with fracture stabilization alone. Both MCS and MCA were significantly greater in the unstable group, and M/S ratio also differed between groups, whereas SCS and MCS–FLSD difference did not. MCA showed high discriminative ability within this cohort, whereas MCS also remained significantly associated with instability but with lower overall discrimination. However, although MCA showed numerically higher discriminative performance than MCS, this difference should be interpreted cautiously because of the limited sample size and the lack of statistical significance in direct AUC comparison. Therefore, our findings support MCA as a potentially useful adjunctive parameter rather than a replacement for established radiographic measures such as MCS.

The role of stress radiographs and MCS in evaluating ankle stability in supination–external rotation (SER) and Weber B-type fractures has been well established [10, 12, 16]. Traditionally, increased MCS on stress radiographs has been regarded as an indirect sign of deep deltoid ligament incompetence, and thresholds in the range of 4 to 5 mm have commonly been cited to suggest instability [10, 12, 14]. At the same time, previous cadaveric and clinical studies have shown that MCS is influenced by ankle position, the method of stress application, fibular displacement, and lateral talar shift [4, 9, 10, 16]. This likely explains why MCS, although clinically useful, does not always correspond perfectly to persistent medial instability after fixation of the lateral malleolus. In the present study, MCS remained significantly greater in the unstable group and retained significance in limited adjusted analyses, which supports its continued value as a practical indicator of instability. Nevertheless, its overall discrimination was lower than that of MCA, and its specificity and positive predictive value were more modest.

Recent work has also emphasized that isolated fibular fractures with suspected deltoid injury should not be assessed solely based on MCS measured on a single radiographic view, and that gravity stress or weight-bearing radiographs may help refine the assessment of medial instability [1, 12–14]. Pitakveerakul et al. reported that an MCS > 4 mm on gravity stress radiographs should raise concern for medial instability, but also noted substantial variability among asymptomatic individuals [14]. James et al. and others have reported that an MCS > 5 mm on external rotation or gravity stress radiographs is associated with deltoid ligament injury; however, the optimal cutoff value and the clinical significance of partial tears remain debated [6, 10, 22]. Consistent with these reports, in our study MCS alone demonstrated only moderate diagnostic accuracy (AUC 0.81) with a relatively modest Youden’s index (0.59), suggesting limited overall discrimination between stable and unstable ankles.

The rationale for evaluating MCA is biomechanically plausible. The deep deltoid ligament is an important restraint to talar external rotation relative to the tibia [7–9], whereas MCS is a linear measurement that may be influenced by factors other than medial-sided ligament insufficiency alone. On this basis, an angular parameter reflecting the relationship between the medial malleolus and the talus on gravity stress radiographs may better capture the rotational component of medial instability. In our cohort, MCA was markedly higher in the unstable group and remained significantly associated with persistent medial instability in exploratory models that included either syndesmotic fixation or posterior malleolar fracture as a covariate. In addition, exploratory sensitivity analyses showed that the association between MCA and persistent medial instability was maintained after excluding patients who underwent syndesmotic fixation, those with posterior malleolar fractures, and those who underwent posterior malleolar fixation. Although these analyses do not eliminate residual confounding, they suggest that the observed association between MCA and persistent medial instability was not solely explained by these subgroups.

The present study also provides additional insight into the relationship between medial clear space widening and fibular displacement. MCS was significantly greater in the unstable group, whereas the MCS–FLSD difference did not differ significantly between groups. In this study, FLSD was used as an exploratory parameter intended to reflect the horizontal displacement associated with the fibular fracture gap on the gravity stress radiograph. Based on this concept, preoperative MCS widening may partly reflect lateral fibular displacement rather than persistent medial instability alone. After reduction and fixation of the lateral malleolus, this horizontal displacement is expected to be largely corrected, which may explain why some ankles with apparent preoperative MCS widening regain medial stability without deltoid ligament repair. In this context, the absence of a significant between-group difference in MCS–FLSD difference supports the possibility that not all preoperative MCS widening represents functionally relevant residual medial instability after fixation.

Syndesmotic injury is another important contributor to ankle mortise instability and must be considered when interpreting medial instability in rotational ankle fractures. In the present study, syndesmotic stability was assessed only after completion of fibular fixation and, when indicated, posterior malleolar fixation. A positive Cotton test prompted trans-syndesmotic screw fixation, and medial instability was then evaluated only after all required bony and syndesmotic stabilization had been completed. This operative sequence was used to minimize the possibility that residual medial widening simply reflected untreated syndesmotic instability rather than persistent medial-sided insufficiency. This issue is particularly relevant because posterior malleolar fixation itself may influence syndesmotic stability and, in some rotational ankle fracture patterns, may reduce the need for separate trans-syndesmotic fixation [23]. In our cohort, however, neither syndesmotic fixation nor posterior malleolar fixation differed significantly between the stable and unstable groups, and subgroup analyses did not suggest that the observed association between MCA and persistent medial instability was explained entirely by these factors.

These syndesmotic findings should nevertheless be interpreted with caution. Our study was limited to surgically treated Weber B fractures, and residual syndesmotic instability requiring fixation was relatively infrequent in this selected cohort. This is clinically plausible, because although Weber B fractures can certainly be associated with syndesmotic injury, syndesmotic disruption is generally more strongly associated with higher fibular fracture patterns and is more consistently expected in Weber C injuries [24–26]. At the same time, recent work has emphasized that Weber B fractures are heterogeneous, and syndesmotic instability can still be present in a meaningful subset of cases [24, 26]. Therefore, our data should not be interpreted as evidence that syndesmotic pathology is unimportant in Weber B fractures, nor do they permit firm conclusions regarding whether deltoid ligament repair is required in fractures with more substantial syndesmotic injury patterns. This question may be better addressed in future studies focused on Weber C fractures or other syndesmotic-dominant rotational injury patterns.

Another important contextual issue is the operative reference used in this study. Our endpoint was not an independently validated measure of deltoid ligament integrity, but rather persistent medial instability on intraoperative fluoroscopic stress testing after completion of fracture and syndesmotic stabilization, with the decision to perform deltoid ligament repair made accordingly. This reflects a pragmatic intraoperative decision-making process that is relevant to real-world surgical planning, but it also retains an element of surgeon judgment. Contemporary arthroscopic literature highlights the limitations of relying exclusively on fluoroscopy for characterizing associated pathology in ankle fractures. Howard et al. reported that arthroscopy performed before open ankle fracture fixation identified intra-articular pathology in 84.2% of cases, with syndesmotic injury being the most common associated lesion [27]. In addition, arthroscopic studies of posttraumatic medial ankle instability have shown that direct visualization can identify medial ligament pathology, including partial injury of the deep deltoid complex, that may not be fully appreciated on conventional imaging alone [28, 29]. Emerging needle arthroscopy techniques may further expand this capability by offering minimally invasive direct assessment of the ankle joint and ligamentous structures, although the current evidence in the ankle remains limited and includes cadaveric feasibility data rather than established clinical validation in acute fracture settings [30]. Therefore, while our fluoroscopy-based intraoperative assessment was clinically pragmatic, it should not be considered equivalent to arthroscopic or MRI-based confirmation of deltoid ligament injury.

The clinical relevance of accurately predicting deltoid ligament injury preoperatively remains a matter of debate. Several comparative studies and meta-analyses have suggested that deltoid ligament repair in Weber B and C fractures may improve radiographic parameters—such as postoperative and final MCS—and reduce malreduction and/or reoperation rates, although improvements in functional outcomes have been less consistent [6, 18, 22, 31]. In our study, clinical outcome scores did not differ significantly between patients who underwent deltoid ligament repair and those who did not, despite clear differences in intraoperative medial stability and radiographic measures. Overall, these findings suggest that radiographic restoration of the ankle mortise and prevention of persistent medial instability are important treatment goals; however, any functional benefit of deltoid ligament repair may require longer follow-up and/or larger cohorts to be detected [6, 18].

These findings have practical implications for surgical decision-making. First, they indicate that a substantial proportion of Weber B ankle fractures with preoperative MCS widening may regain medial stability after fibular fixation alone, underscoring the importance of intraoperative stress assessment before performing deltoid ligament repair [6, 12]. Second, they suggest that preoperative MCA, in conjunction with MCS, may help identify patients at higher risk of persistent medial instability and the intraoperative need for deltoid ligament repair, thereby facilitating preoperative planning and patient counselling. In cases with borderline MCS values but markedly increased MCA, surgeons may anticipate persistent medial instability and be prepared for deltoid exploration and repair; conversely, when MCA is only mildly increased, a more conservative intraoperative strategy may be reasonable.

Several limitations should be considered when interpreting the present findings. First, it was a retrospective single-center study with a relatively small sample size, particularly in the unstable group. Because only 16 patients were classified as having persistent medial instability requiring deltoid ligament repair, the statistical precision of the estimated effect sizes was limited, and the confidence intervals for some exploratory models were wide. For this reason, the adjusted analyses were intentionally restricted to parsimonious exploratory models with only a limited number of covariates. Second, the interval between injury and imaging varied among patients, potentially affecting soft-tissue status and radiographic measurements. Third, gravity stress radiographs were not strictly standardized regarding ankle position or the magnitude of applied stress beyond routine clinical practice, and minor variations in technique could have influenced MCS and MCA values [13, 14]. Fourth, intraoperative medial instability was assessed using fluoroscopic stress testing, and arthroscopic confirmation was not performed, which may have introduced some subjectivity in determining instability and the decision to perform deltoid ligament repair. Fifth, the study cohort was highly selected and consisted of operatively treated Weber B fractures in which deltoid injury was suspected preoperatively; therefore, the present findings should not be generalized to all Weber B fractures or to other fracture patterns without caution. Finally, no formal internal or external validation was performed. The discriminative performance of MCA and the proposed cutoff were derived and tested in the same cohort, and no independent validation sample was available. As a result, the reported AUC values and threshold estimates may be optimistic. Future studies with larger prospective cohorts, formal internal validation procedures, and external validation in independent populations will be necessary to determine the reproducibility, calibration, and generalizability of MCA as a preoperative adjunctive parameter.

Despite these limitations, the present findings suggest that MCA on preoperative gravity stress radiographs may serve as a useful adjunct to MCS for anticipating persistent medial instability and the intraoperative need for deltoid ligament repair in Weber B ankle fractures. The association between MCA and persistent medial instability remained present in limited adjusted analyses and in exploratory sensitivity analyses addressing syndesmotic fixation and posterior malleolar injury. However, given the retrospective design, small number of unstable cases, pragmatic fluoroscopic operative reference, and lack of formal validation, these findings should be regarded as preliminary. Additional prospective studies with larger cohorts and independent validation are required before a specific MCA cutoff can be applied with confidence in broader clinical practice.

## Data Availability

The datasets used and/or analyzed during the current study are available from the corresponding author on reasonable request.

## Abbreviations

MCS: Medial clear space
DLR: Deltoid ligament repair
M/S ratio: Medial–superior clear space ratio
MCA: Medial clear space angle
SCS: Superior clear space
FLSD: Fibular lateral shift distance
VAS: Visual analog scale
FFI: Foot function index
FAOS: Foot and ankle outcome score
AUC: Area under the curve
ROC: Receiver operating characteristic
CI: Confidence interval
NPV: Negative predictive value
PPV: Positive predictive value
